# Relationship of the ORBIT and HAS-BLED scores with Killip class 3-4 in patients with ST-segment elevation myocardial infarction

**DOI:** 10.1097/MD.0000000000014578

**Published:** 2019-02-22

**Authors:** Qing Zhang, Lei Zhou, Hong-Li Cai, Hui-He Lu

**Affiliations:** a Department of Cardiology, The Second Affiliated Hospital of Nantong University, Nantong; b Department of Cardiology, The First Affiliated Hospital of Nanjing Medical University, Nanjing, China.

**Keywords:** HAS-BLED score, Killip classification, myocardial infarction, ORBIT score

## Abstract

Heart failure (HF) complicating ST-segment elevation myocardial infarction (STEMI) is recognized as an ominous complication. The HAS-BLED and Outcomes Registry for Better Informed Treatment (ORBIT) scores are used to assess the bleeding risk in patients with anticoagulated atrial fibrillation. This study aimed to investigate the relationship of the ORBIT and HAS-BLED scores with Killip class 3-4 in patients with STEMI.

639 patients with STEMI were enrolled in this study. The ORBIT and HAS-BLED scores were recorded after admission, and all patients were divided into 2 groups: the Killip class 1-2 and Killip class 3-4 groups. Different clinical parameters were compared. The predictive values of the ORBIT and HAS-BLED scores for Killip classes 3 to 4 were assessed using receiver-operating characteristic (ROC) analyses. Univariate and multivariate logistic analyses were used to evaluate the relationships between variables and Killip class 3-4.

The ORBIT and HAS-BLED scores were higher in the Killip class 3-4 group than in the Killip class 1-2 group (*P* < .05). The areas under the ROC curve of the ORBIT and HAS-BLED scores for predicting the higher Killip classification were 0.818 (95% CI: 0.786–0.847, *P* < .0001) and 0.674 (95% CI: 0.636–0.710, *P* < .0001), respectively. In multivariate logistic analysis, the high ORBIT score was positively associated with Killip classes 3 to 4 after adjustment (odds ratio: 2.306, 95% CI: 1.084–4.911, *P* = .012).

A graded relationship was found in the elevated ORBIT and HAS-BLED scores and Killip classes 3 to 4 in patients with STEMI. The ORBIT score is independently associated with the Killip 3-4 in patients with STEMI.

## 1 Introduction

Heart failure (HF) complicating STEMI is one of the commonest complications. The GRACE study revealed that the proportion of HF patients with acute coronary syndrome (ACS) was 15.6%, and 10.4% of them developed HF after admission.^[1]^ The atrial fibrillation (AF) treatment guideline from the European Society of Cardiology in 2010 first proposed the HAS-BLED scoring system,^[2]^ which is the most extensively used bleeding risk assessment system for anticoagulated patients with AF in the clinical setting. The HAS-BLED score is also used to assess the safety of triple antithrombotic therapy in patients with ACS combined with AF.^[3,4]^ The Outcomes Registry for Better Informed Treatment (ORBIT) score,^[5]^ a new scoring system, is used to assess the bleeding risk in anticoagulated patients with AF. No study has investigated the relationship of the ORBIT and HAS-BLED scores with the Killip classification in patients with STEMI. Therefore, our study aimed to investigate the relationship of the ORBIT and HAS-BLED scores with a high Killip classification among patients with STEMI.

## 2 Methods

### 2.1 Ethics and informed consent

This study was approved by the Ethics Committee of The Second Affiliated Hospital of Nantong University (IRB number: 2018041).

### 2.2 Study population

Six hundred thirty-nine patients with STEMI who were admitted to the Second Affiliated Hospital of Nantong University from January 2017 to June 2017 were enrolled in this study (507 men and 132 women; average age, 66.51 ± 12.91 years). The diagnosis of STEMI was based on the presence of characteristic symptoms of myocardial ischemia, appropriate electrocardiographic changes, and elevation of biomarkers of myocardial necrosis.

### 2.3 Data collection

Age, sex, blood pressure, heart rate, past concomitant diseases (e.g., hypertension, hyperlipemia, diabetes, stroke, renal insufficiency, and old myocardial infarction), smoking history, drug use, Killip class, percutaneous coronary intervention (PCI) treatment, electrocardiographic localizations of myocardial infarction, the time from symptom onset-to-first medical contact (SO-to-FMC), and the ORBIT and HAS-BED scores of all patients were recorded after admission. The troponin I level was immediately assessed on admission. Moreover, fasting venous blood samples were collected the next morning after admission, and the levels of total cholesterol, triglyceride, high-density lipoprotein, low-density lipoprotein, N-terminal pro-brain natriuretic peptide (NT-proBNP), and serum creatinine were measured. Baseline echocardiographic parameters included left ventricular ejection fraction (LVEF), left atrium dimension (LAD), left ventricular end-diastolic diameter (LVDD), and left ventricular end-systolic diameter (LVESD). A total of 431 patients received PCI treatment, all of whom were underwent complete revascularization. Complete revascularization in patients with multiple vessel lesions is performed immediately or in stages, according to the patient's condition.

### 2.4 Killip classification

The Killip class was evaluated as follows according to the classic article^[6]^: class 1, no evidence of HF; class 2, signs indicating mild to moderate degree of HF (e.g., S3 gallop, rales half way up the lung fields, or elevated jugular venous pressure); class 3, pulmonary edema, and class 4, cardiogenic shock or hypotension.

### 2.5 The ORBIT score

The ORBIT score^[5]^ was developed from the ORBIT registry and calculated as follows: 1 point each for age ≥75 years, insufficient kidney function (glomerular filtration rate <60 ml/min/1.73 m^2^), and treatment with any anti-platelet agent; and 2 points for a history of bleeding and reduced hemoglobin level/anemia (<13 mg/dl for men and <12 mg/dl for women or hematocrit <40% for men and <36% for women).

### 2.6 Statistical analysis

Normally distributed continuous variables are presented as mean ± standard deviation, whereas those conforming to a skewed distribution are expressed as a median (25th percentile–75th percentile). The enumeration data are presented as percentage or frequency. The independent-samples *t* test, and Mann–Whitney *U* test and χ^2^ test were used to compare the measurement and enumeration data of the 2 groups, respectively. The predictive values of the ORBIT and HAS-BLED scores for Killip class 3-4 in patients with STEMI were determined using receiver-operating characteristic (ROC) analyses. Univariate and multivariate logistic analyses were used to evaluate the relationship between variables and a higher Killip classification. Variables that had a *P* value <.1 in the univariate analysis were used in a multivariable logistic model to determine their independent association with Killip class 3-4. Data were analyzed using SPSS 17.0 (SPSS Inc., Chicago, IL) and Med-Calc (version 11.2.1; MedCalc, Mariakerke, Belgium). A *P* value <.05 was considered statistically significant.

## 3 Results

A comparison of the baseline characteristics between the 2 groups showed that age, diabetes, heart rate, SO-to-FMC, the HAS-BLED and ORBIT scores, NT-proBNP level, serum creatinine level, troponin I level, LVDD, and LVESD were higher in the Killip 3-4 group than in the Killip 1-2 group (*P* < .05), whereas PCI treatment, systolic blood pressure, ACEI or ARB (%), Beta-blockers (%) and LVEF were lower in the Killip 3-4 group than in the Killip 1-2 group (*P* < .05; Table 1).

**Table 1:**
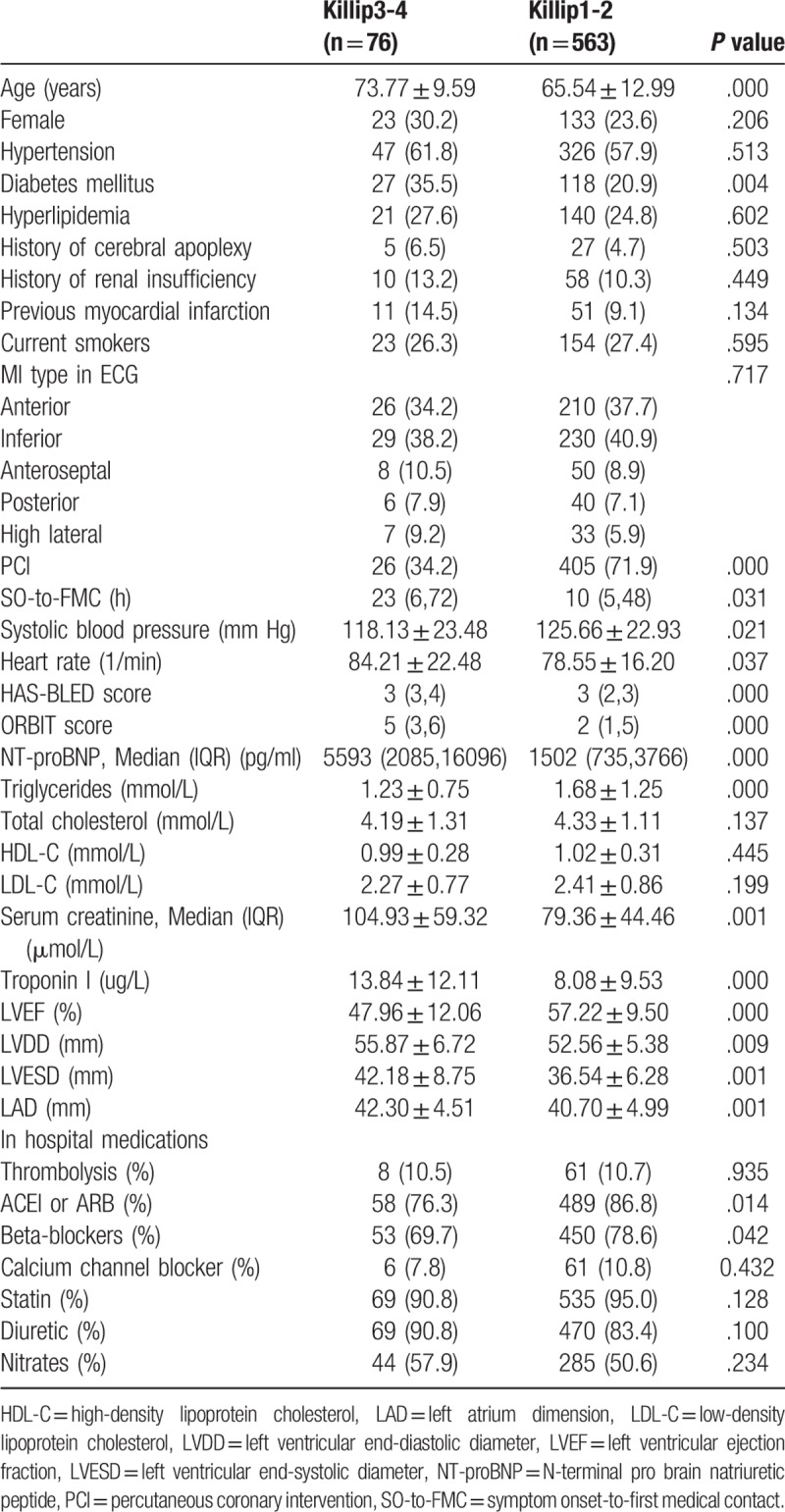
Comparison of the general clinical information between the 2 groups.

**Figure 1:**
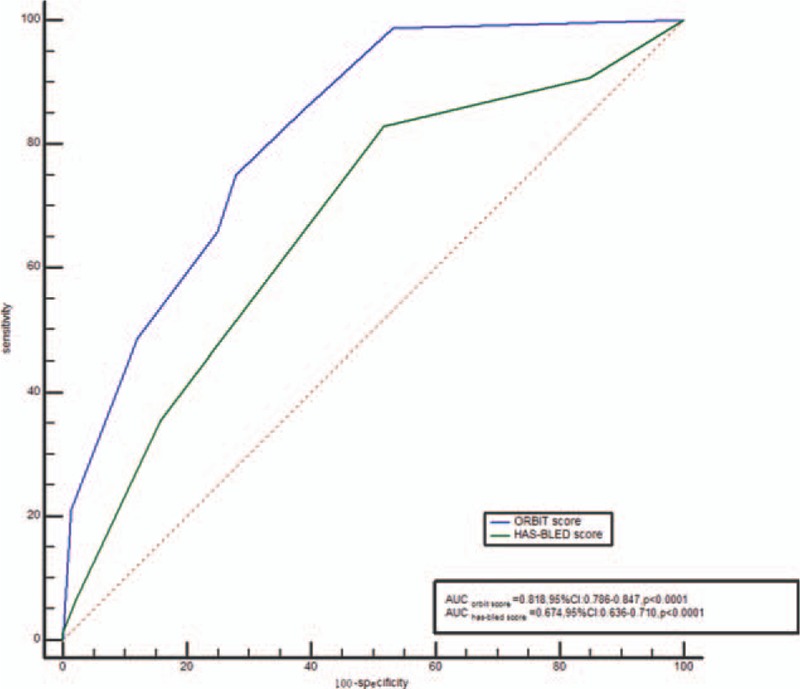
Diagnostic accuracy for killip class 3-4.

The area under the ROC curve of the HAS-BLED score for predicting Killip 3-4 was 0.674, with a cut-off level of 2 (95% CI 0.636–0.710), sensitivity of 82.9%, and specificity of 48.2% (*P* < .0001). The area under the ROC curve of the ORBIT score for predicting Killip 3-4 was 0.818, with a cut-off level of 3 (95% CI 0.786–0.847), sensitivity of 75.0%, and specificity of 72.1% (*P* < .0001; Fig. 1).

Variables that had a *P* value <.1 in the univariate analyses were used in a multivariate logistic regression analysis model. After forward step-wise multivariate analyses, variables for inclusion in the multivariate analyses were age, diabetes, heart rate, SO-to-FMC, the HAS-BLED and ORBIT scores, NT-proBNP level, serum creatinine level, troponin I level, LVDD, LVESD, PCI treatment, systolic blood pressure, ACEI or ARB (%), Beta-blockers (%), and LVEF. In the multivariate analysis model, the high ORBIT score was significantly associated with a high Killip class in patients with STEMI after adjustment (odds ratio: 2.306, 95% CI: 1.084–4.911, *P* = .012) (Table 2).

**Table 2:**
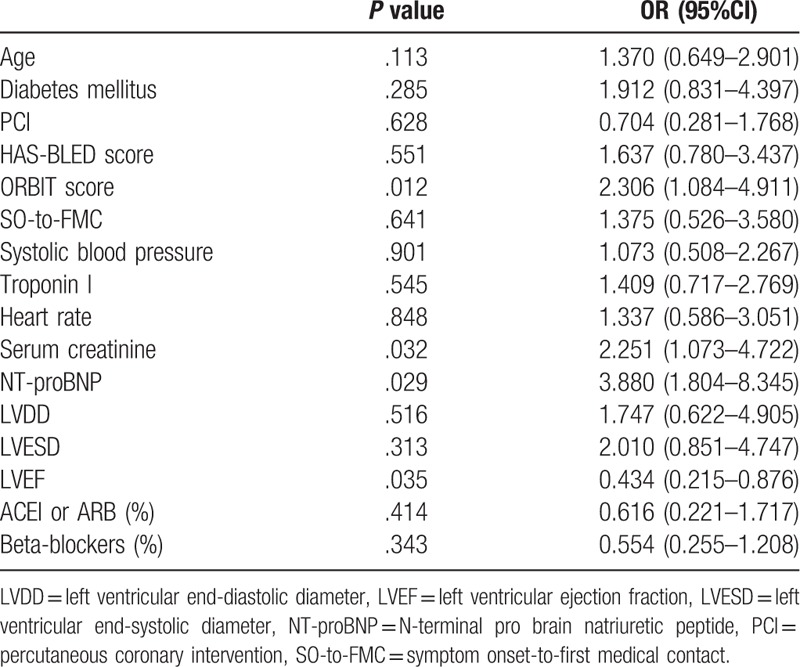
Results of multivariate analyses for Killip 3-4.

## 4 Discussion

Our study found that the Killip class 3-4 group had higher ORBIT and HAS-BLED scores than the Killip class 1-2 group. The ROC curve showed that the ORBIT score had better predictive value for a high Killip classification than the HAS-B-LED score. Multivariate logistic regression analysis indicated that the increased ORBIT score is independently associated with Killip class 3-4.

Acute myocardial infarction remains a leading cause of death worldwide.^[1]^ In addition, acute HF (AHF) has been widely recognized as a marked complication of STEMI, which is linked with dismal short-term and long-term prognoses.^[7–9]^ At present, most existing studies are limited to reperfusion therapies and the inclusion of heterogeneous populations in terms of the types of acute coronary syndromes.^[1,10–12]^ The Killip classification is a predictor of short- and long-term prognoses for patients with ACS.^[13]^ A previous study showed that the corresponding mortality rates for Killip classes 1, 2, 3, and 4 were 6%, 17%, 38%, and 81%, respectively.^[6]^ Recently, the ORBIT score, a new bleeding prediction score, has been developed from a large observational cohort of patients with AF,^[5]^ and it has been proposed as a simple bedside score. Some studies^[14,15]^ found that the HAS-BLED score can also be used to predict the prognosis of non-AF patients receiving PCI. However, no data have indicated the relationship among the HAS-BLED, ORBIT scores, and Killip classification in patients with STEMI.

We believe that the relationship of the ORBIT score with HF after STEMI can be explained by the following theory. The incidence of renal dysfunction is high in patients with HF. Worsening renal function occurs frequently among hospitalized patients with HF and is associated with significantly worse outcomes. The mortality and re-hospitalization rate of HF patients with renal insufficiency have remarkably increased compared with those of HF patients with normal renal function.^[16–18]^ A study found that ACS patients with poor renal function tended to have a higher Killip class.^[19]^ HF is also closely related to age.^[20]^ Most people aged ≥65 years are hospitalized for HF.^[21]^ Moreover, increased age has been identified as an independent risk factor for mortality in patients with HF.^[22–24]^ Decreased hemoglobin and reduced hematocrit levels are all manifestations of anemia. One-third of all patients with HF have anemia.^[25,26]^ Further, anemia is common and associated with an increased risk of mortality and hospitalizations in a wide range of populations with HF.^[27]^ Age, decreased hemoglobin level, and insufficient kidney function are important factors that affect the ORBIT score. Therefore, STEMI patients with a higher ORBIT score may be associated with poorer renal function, older age, and higher anemia incidence. Therefore, patients with a higher ORBIT score have poorer cardiac function.

## 5 Conclusion

The ORBIT score is independently associated with a high Killip classification in patients with STEMI.

## Author contributions

**Conceptualization:** Hui-He Lu.

**Data curation:** Hong-Li Cai.

**Supervision:** Qing Zhang.

**Visualization:** Lei Zhou.

**Writing – review & editing:** Qing Zhang.
